# Evidence of goose circovirus identified in Muscovy ducks

**DOI:** 10.3389/fvets.2026.1747242

**Published:** 2026-03-04

**Authors:** Mengyan Zhang, Youlin Chen, Zhongjun Tian, Yu Huang, Chunhe Wan, Guangwen Yin, Ye Chen

**Affiliations:** 1 College of Animal Sciences, Fujian Agriculture and Forestry University, Fuzhou, China; 2 Fujian Key Laboratory for Avian Disease Control and Prevention, Institute of Animal Husbandry and Veterinary Medicine, Fujian Academy of Agricultural Sciences, Fuzhou, China

**Keywords:** analysis, cross-species transmission, genome, goose circovirus, Muscovy duck

## Abstract

Goose circovirus (GoCV) has been found in waterfowl more than 20 years, causing considerable economic loss worldwide. In the present study, goose circovirus (designated DuGoCV) was identified in Muscovy ducks in China, with complete genomes were sequenced and analyzed. The genetic evolutionary characteristics and recombination patterns of different genotypes were examined. The DuGoCV strain shared genome sequence identities of 89.2–95.9% with the GoCV reference strains and 67.0–67.1% with the DuCV reference strains. Phylogenetic analysis revealed that the DuGoCV strain belongs to GoCV-III. These findings suggest possible cross-species transmission of GoCV between geese and Muscovy ducks, providing important insights for the prevention and control of GoCV infection.

## Highlights

- We first report goose circovirus (designated the DuGoCV strain) identified in Muscovy duck.
- A potential recombination event was identified for DuGoCV.
- Our data provide valuable insights for understanding the genetic diversity and cross-species transmission potential of GoCV.

## Introduction

According to the latest classification by the International Committee on Taxonomy of Viruses (ICTV), the family Circoviridae comprises two genera: Circovirus and Cyclovirus. GoCV belongs to the genus *Circovirus* within the family *Circoviridae* (1). The GoCV genome consists of covalently closed circular, single-stranded DNA. The GoCV genome contains four open reading frames (ORFs): ORF V1 (72–953 bp) and ORF V2 (1612–1725 bp) located on the sense strand and ORF C1 (1761–1008 bp) and ORF C2 (426–127 bp) on the complementary strand. Current research primarily focuses on ORF V1 and ORF C1. ORF V1 encodes the replication protein (Rep), which is involved in viral DNA replication, while ORF C1 encodes the capsid protein (Cap), the major structural protein of the virus.

GoCV was first identified in goose flocks in Germany in 1999 (2) and has subsequently been reported in various regions (3–7). Recently, cross-species infection cases have also emerged (8, 9). The overall prevalence of GoCV infection in ducks is relatively low and exhibits clear spatial clustering. Once introduced into a susceptible duck population, the virus may cause a high proportion of infections. Currently, GoCV infection exhibits a broad prevalence in goose populations (3), with its epidemiological patterns influenced by geographic location, while bird migration and poultry trade are also considered contributing factors (6). Presently, although there is no unified classification standard for GoCV, multiple studies have categorized the virus based on its genetic evolution (GoCV-I, GoCV-II, and GoCV-III) (10, 11). Infection with GoCV in poultry primarily manifests clinically as developmental delay, disordered feathering, emaciation, and lethargy. In severe cases, feather loss and feather follicle necrosis may occur. The presentation of these clinical signs may be associated with factors such as age at infection, viral virulence, and secondary infections (12, 13). Studies have also revealed that coinfection with GoCV and other viruses can increase mortality rates in affected poultry (8).

In this study, using whole-genome sequencing, comparison with reference sequences, phylogenetic analysis, and recombination analysis, we report the first case of GoCV infection in Muscovy ducks. These findings provide valuable insights for understanding the genetic diversity and cross-species transmission potential of GoCV.

## Materials and methods

### Sample collection

A total of 429 tissue samples were collected from diseased birds exhibiting clinical signs, including disordered feathering, diarrhea, and growth retardation, in Fujian, Jiangxi, Anhui, and Shandong provinces. Of the 429 tissue samples, 83 were obtained from Muscovy ducks. All samples were collected from individual birds, with ages ranging from 1 to 300 days. Samples were collected during necropsy, where liver tissues were homogenized and prepared for testing. The tissue homogenates were subjected to three freeze-thaw cycles, followed by centrifugation at 8000×g for 10 min at 4 °C. The supernatant was then collected for nucleic acid extraction. Viral DNA was extracted from the tissues using a commercial kit (TransGen Biotech, Beijing, China) according to the manufacturer’s instructions and stored at −80 °C. PCR screening was performed on viral DNA using universal primers targeting common pathogens such as waterfowl circoviruses (duck circovirus and GoCV) (14), duck adenovirus 3 (15), waterfowl parvoviruses (Muscovy duck parvovirus and goose parvovirus) (16), and Tembusu virus (17). The single GoCV-positive sample identified in this batch was collected from a farm in Fujian Province.

### PCR amplification and sequencing

Using the extracted DNA as a template, sample detection was initially performed via PCR with specific primers according to a previously described method (14). The sequences of these primer pairs were P1814f: 59-ATA TTA CCG GCG C(C/T)T GTA-39 and P248r: 59-TCA GGA ATC CCT G(A/C)A GGT GA39. This primer pair amplified a 256 bp fragment from GoCV and a 228 bp fragment from DuCV. Positive samples were then selected for full-genome amplification via a universal primer pair (11). The PCRs were carried out in a total volume of 25 μL, containing 15 μL of DreamTaq Green PCR Master Mix, 2 μL of template DNA, 2 μL of primer (1 μL each), and RNase-free H_2_O added to a final volume of 25 μL. The amplification conditions were as follows: initial denaturation at 94 °C for 5 min; 35 cycles of denaturation at 94 °C for 30 s, annealing at 54 °C for 30 s, and extension at 72 °C for 1 min; and a final extension at 72 °C for 7 min. PCR was carried out in a TaKaRa PCR Thermal Cycler Dice™ Gradient system (TP600, Beijing, China). The target PCR products were purified using an EasyPure® PCR Purification Kit (TransGen Biotech, Beijing, China) and then cloned using a pEASY®-T1 Simple Cloning Kit (TransGen Biotech, Beijing, China) according to the manufacturer’s instructions. After transformation of Trans10 Chemically Competent Cells (TransGen Biotech, Beijing, China) with these constructs, three positive clones for each PCR product were sequenced in both directions using an ABI 3730XL DNA Analyzer (Applied Biosystems, CA, United States) at Sangon Biotech Co. (Shanghai, China).

### Sequence analysis and phylogenetic analysis

The obtained sequencing results were assembled and consolidated via the SeqMan program within the DNAStar software package (DNASTAR Inc., WI, United States). The resulting complete genome sequences were aligned and analyzed alongside a dataset of 49 reference strains from the GenBank database, comprising 2 DuCV and 47 GoCV genomes. Sequence homology analysis for GoCV and DuCV was conducted via the MegAlign program of the Lasergene 7.0 package. A phylogenetic tree was constructed with the Phylogeny function in MEGA 12 software, employing the maximum likelihood method based on the default model with 1,000 bootstrap replicates for evolutionary analysis. Recombination events in the DuGoCV strain and the reference strains were analyzed via RDP5.0.

## Results and discussion

### Sample screening

Horizontal and vertical transmission of DuCV have been documented (18, 19), and it has been confirmed that DuCV can infect geese (9). However, the modes of GoCV transmission remain unverified and require further investigation. While existing studies have indicated that GoCV can infect ducks (9), our study is the first to identify GoCV infection in Muscovy ducks, providing further evidence for the cross-species transmission potential of GoCV. Therefore, the impact of GoCV infection on the growth and health of poultry warrants greater attention from researchers and poultry breeders.

### DNA alignment and similarity analysis

**Figure 1:**
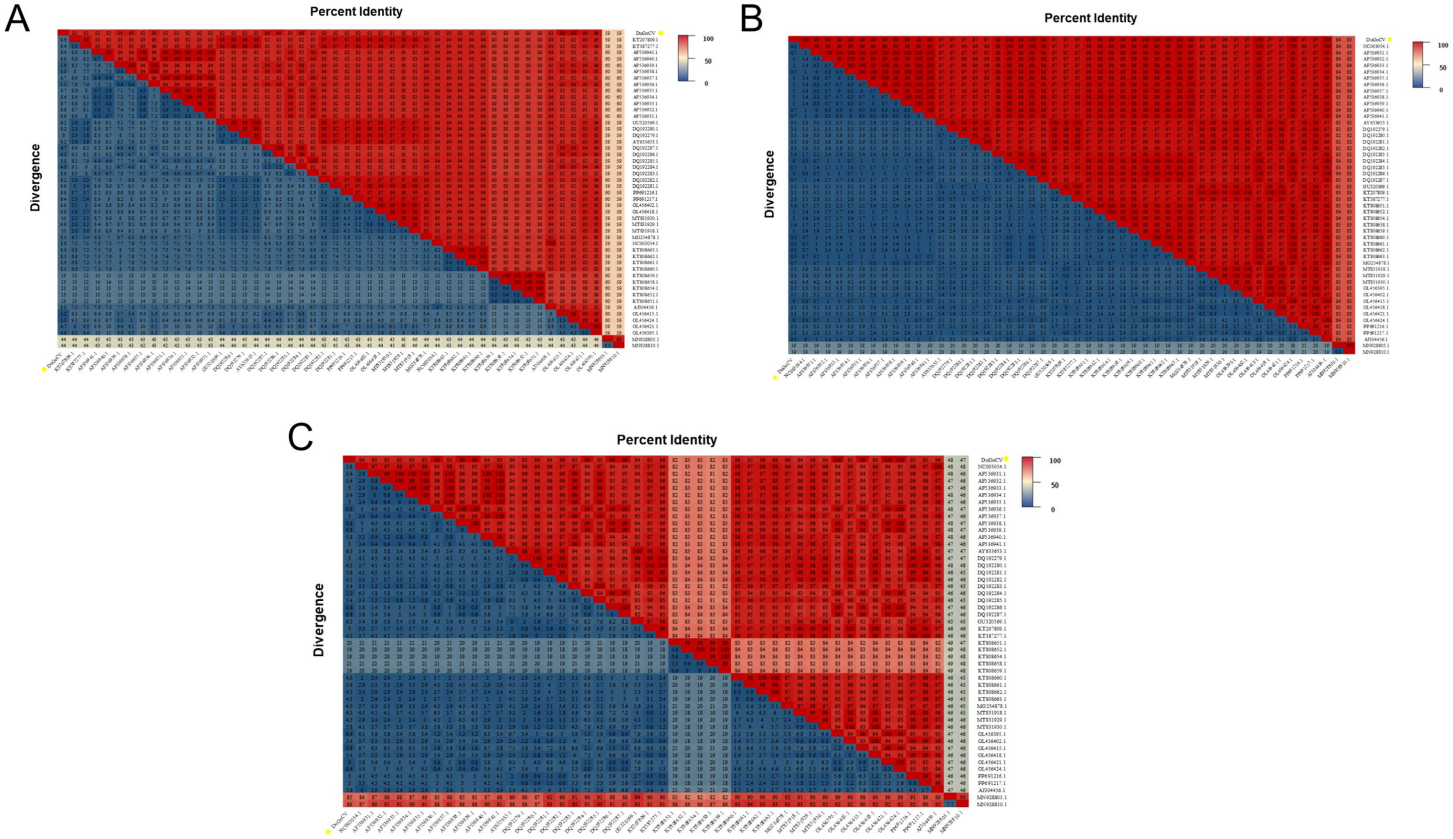
Heatmap of genome-wide similarity analysis of the DuGoCV and reference strains obtained in this study. **(A)** Shows the results of whole-genome amino acid homology analysis. **(B)** Shows the results of the Rep protein amino acid homology analysis. **(C)** Shows the results of Cap protein amino acid homology analysis.

The complete genome sequencing results revealed that the DuGoCV strain has a total genome length of 1,821 nt. Homology analysis was performed by comparing the DuGoCV sequence with 47 GoCV and 2 DuCV complete genome sequences published in GenBank. The results (Figure 1) indicated that DuGoCV-1 shared genome-wide sequence identities of 89.2–95.9% with GoCV strains (KT207809.1 to OL456415.1) and 67.0–67.1% with DuCV strains (MN928805.1 to MN928810.1).

The amino acid sequence similarity of the Rep protein between DuGoCV strains and GoCV reference strains ranged from 97 to 100%, while it was between 83 and 84% compared to DuCV reference strains. For the Cap protein, the similarity between DuGoCV and GoCV reference strains ranged from 82 to 100%, whereas it was only 47 to 48% relative to DuCV reference strains. These results indicate that the Rep protein sequence is more conserved, while the Cap protein exhibits a higher degree of variability.

### Phylogenetic analyses

**Figure 2:**
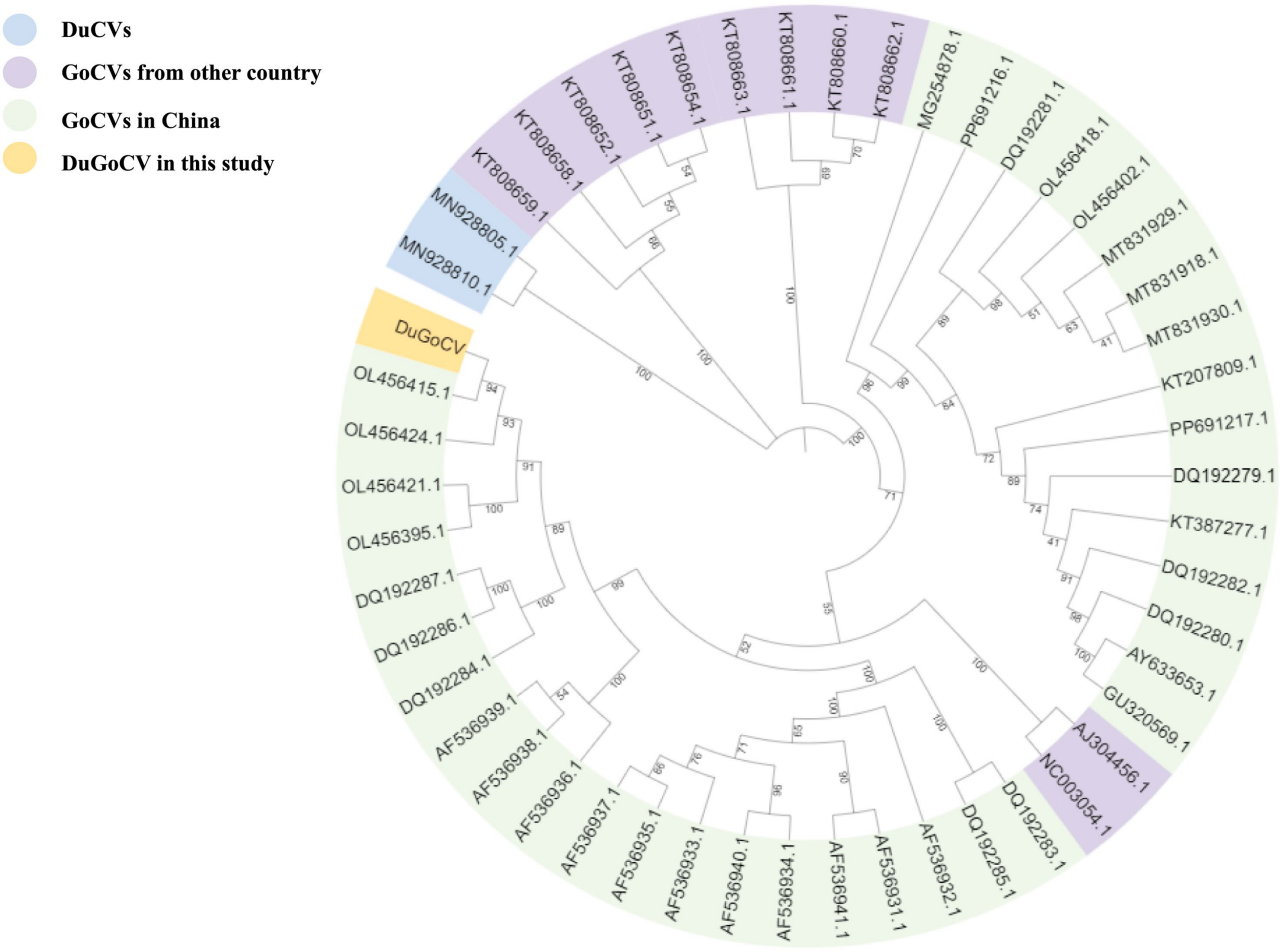
Phylogenetic tree constructed from whole-genome sequences of GoCV and duck circovirus.

A phylogenetic analysis was performed on the basis of the complete genome sequences of the DuGoCV strain obtained in this study, along with 47 GoCV and 2 DuCV reference strains from the GenBank database. The results (Figure 2) showed that the DuGoCV strains clustered within the evolutionary branch of GoCV viruses. DuGoCV is most closely related to a goose-origin GoCV strain from Foshan, Guangdong, in 2022 (OL456415.1). Notably, GoCV strains from the same geographical location were typically concentrated within the same evolutionary branch, suggesting a potential correlation among the transmission, genetic evolution, and geographical distribution of GoCV. The isolate identified in this study showed a close relationship to GoCV strains previously reported in Guangdong, China. This may be attributed to frequent poultry trade between Guangdong province—the largest goose breeding and consumption area in China—and other regions, which likely facilitated viral dissemination.

### Recombination analysis

**Figure 3:**
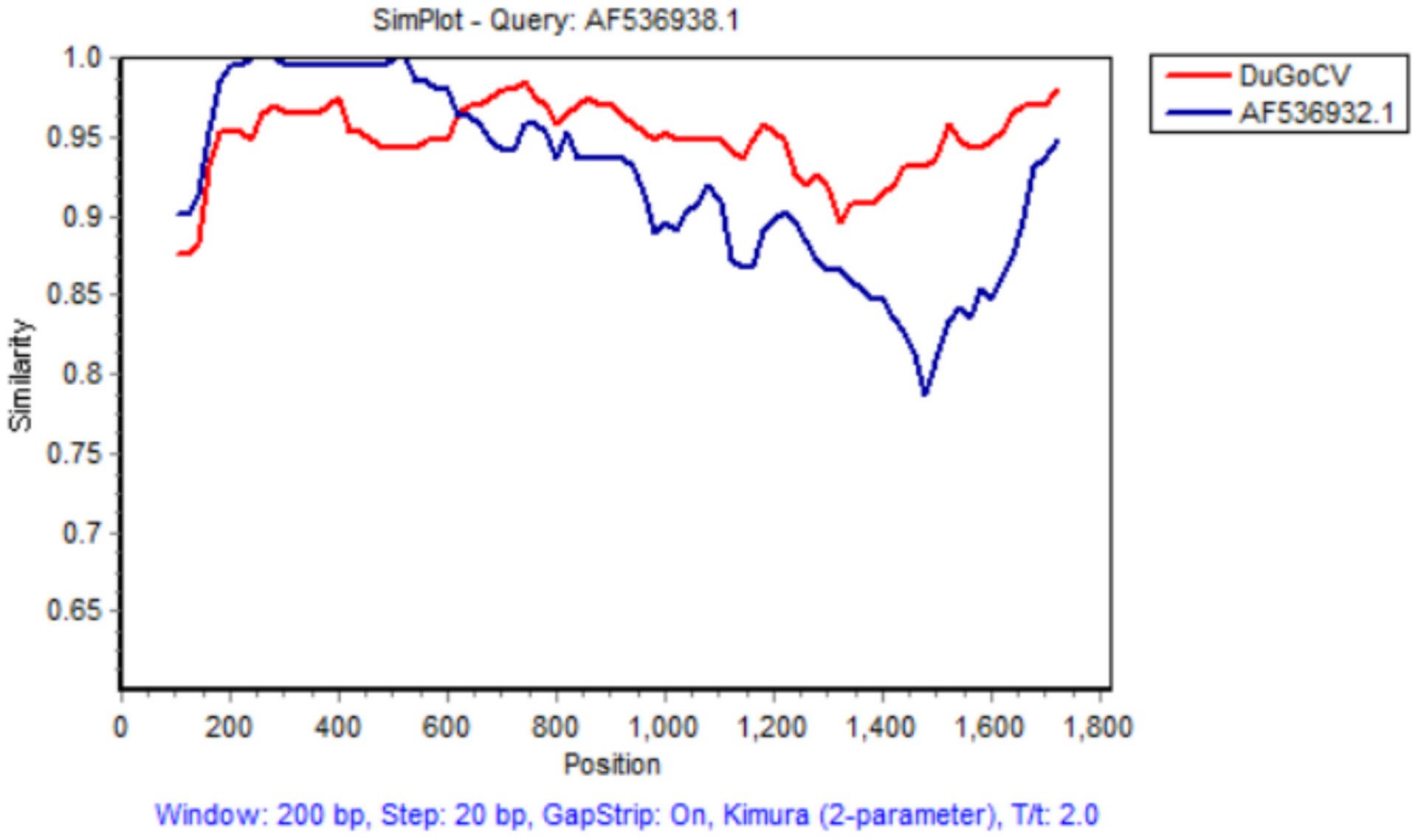
Recombination events for the DuGoCV strain were calculated via Simplot software.

Recombination is a significant driving force in circovirus evolution. Studies indicate that frequent recombination events occur in GoCV in nature (7). However, because recombination can obscure evolutionary relationships among viruses, phylogenetic trees constructed solely based on partial gene or complete genome sequences may not accurately reflect true evolutionary history. Research from Poland identified that most GoCV strains circulating in domesticated and wild geese were recombinants, with recombinant fragments comprising up to 50% of the entire genome (10). Furthermore, a study in China also reported recombination events in GoCV identified in duck populations (9). Recombination analysis enables precise identification of parental strains, recombination breakpoints, and the resulting chimeric genome structures, thereby elucidating how novel genetic variations arise through the exchange of genetic material. Moreover, as recombination can lead to antigenic drift and changes in virulence, recombination analysis helps trace the origin of variations in these critical genomic regions. This is of significant importance for early warning of emerging infectious diseases and for assessing biosecurity risks. Analysis using multiple algorithms in RDP5.0 (RDP, GENECONV, BootScan, Maxchi, Chimera, SiScan, and 3 seq) revealed that DuGoCV was not identified as a recombinant. However, a potential recombination event was detected in the TW8 strain (AF536938.1) from Taiwan, China, with the DuGoCV strain obtained in this study serving as its major parent and the TW2 strain (AF536932.1) implicated as a putative minor parent. Regarding this recombination event, a total of seven algorithms—RDP, GENECONV, BootScan, Maxchi, Chimera, SiScan, and 3 seq—were employed for prediction. The results obtained from all seven algorithms were completely consistent, each indicating a significant positive signal for recombination. A potential recombination event was identified at nucleotide position 635. Simplot software confirmed this potential recombination event, yielding results consistent with those from RDP5.0 (see Figure 3).

Current GoCV infection has been characterized by a complex epidemiological situation, with successive studies identifying cross-species transmission events. In 2013, Shehata et al. (8) detected one GoCV-positive sample in an ornamental duck. Subsequently, in 2024, Xu et al. (20) identified GoCV in samples collected from two separate duck farms. These findings indicate that duck flocks are experiencing not only DuCV infections but also cross-species infections by GoCV and even coinfections with both GoCV and DuCV. Furthermore, the absence of a suitable cell culture system for isolating and propagating GoCV poses a significant challenge for in-depth investigation into its pathogenic mechanisms.

Circoviruses have been detected not only in poultry and livestock but also in a wide range of wildlife, including waterfowl, as well as in human and chimpanzee fecal samples. The Rep protein of GoCV shares high amino acid sequence homology with Rep proteins from other animal circoviruses. Together, these observations indicate a potential for cross-species transmission in this group of viruses. Furthermore, recombination occurs frequently during GoCV spread. A molecular epidemiological study of GoCV in southern China revealed extensive genetic diversity in its genome and identified multiple recombination events. The combination of cross-species transmission capability and recombination ability drives a “transmission-recombination-retransmission” cycle in GoCV epidemiology. Cross-species transmission enables different viral strains to meet in a broader host reservoir, thereby increasing opportunities for recombination. In turn, recombinant strains with novel characteristics may further expand their host range or enhance their fitness. This cycle substantially increases the genetic complexity and evolutionary rate of GoCV.

In summary, this study applied PCR technology to conduct an epidemiological investigation of circovirus in Muscovy ducks and successfully identified one viral strain. Complete genome sequencing and genomic analysis confirmed this strain as GoCV-III. This study reports the first case of GoCV infection in Muscovy ducks, indicating the occurrence of cross-species transmission of the virus. These findings provide valuable insights for future research into the mechanisms of GoCV cross-species infection and the development of prevention and control strategies.

## Data Availability

The data presented in the study are deposited in the National Center for Biotechnology Information, accession number PZ017942.
